# The role of home-based math activities in developing mathematical reasoning skills in preschool children: a pilot randomized controlled trial

**DOI:** 10.3389/fpsyg.2026.1903843

**Published:** 2026-09-01

**Authors:** Bayram Deleş, Neriman Aral

**Affiliations:** 1 Department of Child Development, Faculty of Health Sciences, Tarsus University, Mersin, Türkiye; 2 Department of Child Development, Faculty of Health Sciences, Ankara University, Ankara, Türkiye

**Keywords:** early childhood education, home-based education, intervention, mathematical reasoning, preschool mathematics

## Abstract

**Background:**

Early mathematical reasoning skills are important predictors of later academic achievement. Although home-based learning environments contribute to early mathematics development, structured home-based intervention programs specifically targeting mathematical reasoning skills remain limited.

**Methods:**

The study employed a pilot randomized pretest–posttest control group design to evaluate the HBMR program. The study sample consisted of 20 preschool children (10 in the experimental group and 10 in the control group) selected using a stratified random sampling procedure. Following pre-test assessments, participants were randomly assigned to either the experimental or control group. The HBMR program was implemented over 5 weeks through ten structured home sessions, including reasoning-based activities such as matching, classifying, comparing, sequencing, and basic number operations. As the data did not meet normality assumptions, non-parametric analyses (Mann–Whitney U and Wilcoxon signed-rank tests) were conducted. Effect sizes were calculated using the formula r = Z/√N.

**Results:**

Wilcoxon signed-rank test results revealed significant improvements in the experimental group in measurement (*Z* = −2.684, *p* = 0.007), data analysis (*Z* = −2.831, *p* = 0.005), and overall mathematical reasoning scores (*Z* = −2.812, *p* = 0.005), with large effect sizes (*r* = 0.85–0.90). No statistically significant improvements were observed in the control group. Mann–Whitney U test results indicated that the experimental group’s post-test scores were significantly higher than those of the control group (*p* < 0.05).

**Conclusion:**

The findings indicate that children who participated in the HBMR program showed greater improvements in mathematical reasoning skills than children in the control group.

## 1 Introduction

Mathematics is a common language across societies worldwide and a fundamental necessity in daily life. Scientists define mathematics as a powerful tool that must be learned to understand and organize the world (Somuncu and Aslan, 2022). In this context, mathematical thinking is a process that should be instilled in children during early childhood, enabling the development of higher-level mental skills such as cause-and-effect relationships and reasoning, and most importantly, covering the fundamentals of mathematics (Vanluydt et al., 2021). Especially today, mathematical thinking, problem-solving, and reasoning skills are among the most important competencies of the 21st century (Deleş et al., 2025).

One of the fundamental skills in mathematics is reasoning, defined as considering all available data when solving a problem or reaching a logical conclusion (Çelik et al., 2025). Reasoning is the process of filtering information obtained from stimuli in one’s mind to reach a specific decision (Ergül, 2014; Mues et al., 2022). Mathematical reasoning is reasoning specific to mathematics and involves learning mathematical concepts, properties, and operations by going beyond routines and proving interrelated facts through reasoning (Hallumoğlu et al., 2023; Skwarchuk et al., 2014). This process is explained by two basic approaches: induction, which leads to general conclusions, and deduction, which yields definite conclusions (Ergül and Artan, 2015). In this context, mathematical reasoning is a mental skill that should be encouraged from preschool education onwards to help children understand mathematics more deeply by justifying solutions, developing ideas, and making sense of events (Deleş et al., 2025; Hornburg et al., 2021; Hallumoğlu et al., 2023).

The preschool years are a critical developmental period during which the foundations of children’s mathematical skills are laid, and their future academic success is shaped (Aral et al., 2011; Gür and Kölemen, 2024). However, the final years of the preschool period—60–72 months—represent a significant developmental transition during which children begin to transform their basic mathematical knowledge into higher-level mathematical thinking processes. During this period, children do more than count objects; they begin to use skills that form the basis of mathematical reasoning—such as matching, classifying, comparing, ordering, pattern recognition, and explaining relationships between quantities—in a more systematic manner (Greenes et al., 2004; Çelik and Kandır, 2013). It has been reported that early mathematical skills strongly predict later mathematical success and that mathematical proficiency, particularly during the ages of five to six, is a key determinant of elementary school achievement (Duncan et al., 2007; Watts et al., 2014; Clements and Sarama, 2025).

Recent research shows that mathematical reasoning in five- and six-year-old children is based not only on number sense but also on the integration of various cognitive and mathematical processes. Children in this age group can classify objects based on different characteristics, compare quantities, form patterns, associate number symbols with quantities, and justify their solutions (Jordan et al., 2022; Träff et al., 2023). Furthermore, symbolic number comparison, number line estimation, and working memory have been reported as important predictors of mathematical proficiency (De Smedt et al., 2013; Passolunghi and Costa, 2016). A recent study by Mera et al. (2026) involving children with an average age of 63 months also demonstrated that verbal short-term memory, working memory, symbolic comparison, and number line estimation significantly explain early mathematical competence. These findings indicate that the 60–72-month period is developmentally critical for structured interventions aimed at supporting mathematical reasoning.

The development of children’s mathematical reasoning skills depends not only on the presentation of mathematical content but also on the structuring of this content in a way that is appropriate for children’s developmental levels of thinking. According to Clements and Sarama’s Learning Trajectories approach, early mathematics instruction consists of the targeted mathematical outcomes, the developmental progression steps children follow to achieve these outcomes, and the instructional activities that support this progression. In this approach, basic skills such as matching, classifying, comparing, ordering, pattern recognition, and measuring are not viewed as independent activities but rather as developmental steps that lead children toward higher-level mathematical thinking and reasoning (Sarama and Clements, 2017; Clements and Sarama, 2021; Clements and Sarama, 2025). Piaget’s theory of cognitive development also explains this developmental progression. According to Piaget (1970), between ages four and seven, children shift from intuitive thinking toward more logical associations; they use cognitive processes such as comparison, classification, and ordering more systematically. This developmental change constitutes one of the theoretical foundations explaining why mathematical reasoning becomes evident in the final year of the preschool period.

To understand a child’s development, it is necessary to understand not only the individual but also the relationship systems surrounding them. In this context, Bronfenbrenner's (1977) Ecological Systems Theory defines the intertwined layers that influence an individual’s development and focuses on the interactions within these layers. One of these layers, the mesosystem, encompasses the interactions and relationships between two or more microsystems in which the child actively participates (Bronfenbrenner, 1986). In this context, the interaction between the family and the school forms the most fundamental mesosystem structures (Tudge et al., 2009). When the relationships between these microsystems are disrupted, children struggle to explore other parts of their environment (Eslek and Yılmaz Irmak, 2018). In this context, families communicating with teachers about their children’s development and academic success and being involved in their children’s learning processes at home indicate a healthy bond within the mesosystem (Tomasetto et al., 2015). From this perspective, supporting mathematical reasoning skills, one of the child’s fundamental developmental areas, in the home environment is an important factor during the 60–72-month period when the foundations of mathematical reasoning skills are laid (Deleş et al., 2025; Tay et al., 2021).

Home-based education is a support model provided by a professional visitor in the family’s home environment. This model provides parents with materials appropriate to the child’s developmental needs and aims to make the home environment more nurturing (Cahoon et al., 2023). Supporting parenting skills and improving the home environment are effective in children’s development (McConkey, 2024). Such programs also play an important role in children’s development, especially those in preschool education. Research shows that home-based interventions positively affect cognitive, language, and academic development, particularly during the preschool years (Niklas and Schneider, 2017). Studies conducted in Türkiye and internationally also report that home-based programs increase parental involvement and lead to significant improvements in children’s developmental outcomes (Anders et al., 2012; Bachman et al., 2020; Çelik et al., 2025; Daucourt et al., 2021; Elbir, 2023; Eslek and Yılmaz Irmak, 2018; Gür and Kölemen, 2024; Hornburg et al., 2021; Kalogeropoulos et al., 2021; Niklas et al., 2016; Panaoura, 2021; Sonnenschein et al., 2020; Tay et al., 2021).

Recent research in early mathematics indicates that not only the implementation of home-based programs but also the quality of the home mathematics environment plays a decisive role in children’s mathematical development. It has been reported that mathematical interactions occurring in daily life—such as counting, comparing, classifying, sequencing, pattern recognition, measuring, and problem-solving—significantly support children’s early mathematics skills (Elliott and Bachman, 2018; Hornburg et al., 2021; Skwarchuk, 2009; Sonnenschein et al., 2020; Susperreguy and Davis-Kean, 2016). However, research indicates that it is not only the frequency but also the quality of mathematics activities conducted at home that is important. When parents ask children open-ended questions, use mathematical language, and encourage children to explain their problem-solving approaches, they strengthen mathematical learning. Indeed, Purpura et al. (2021) demonstrated that a structured mathematical language intervention for parents significantly improved preschool children’s mathematical knowledge and skills.

In contrast, many parents tend to limit mathematics primarily to counting and performing arithmetic operations; they are unable to sufficiently support higher-order mathematical processes such as comparison, classification, pattern recognition, reasoning, and mathematical explanation in the home environment (Casey et al., 2018; Puccioni, 2018; Silver et al., 2021). Similarly, although parents are aware of the importance of math activities, they report not feeling sufficiently equipped to support their children’s mathematical thinking (Ergel and Aydoğan, 2021). Furthermore, parents’ attitudes toward mathematics, their math anxiety, and their past experiences can also influence the development of children’s mathematical reasoning skills (Bilgen and Akman, 2021; DeFlorio and Beliakoff, 2015; Deleş and Aral, 2025; Meyer et al., 2011; Mues et al., 2022). When these findings are considered together, it becomes clear that there is a need for developmentally structured, home-based intervention programs that support children’s mathematical reasoning by guiding their parents.

Based on these considerations, the present study was designed to experimentally examine the effectiveness of structured home-based activities aimed at supporting preschool children’s mathematical reasoning skills while also guiding parents in providing cognitively stimulating learning experiences at home. The program was informed by constructivist principles (Çetinkaya, 2023), emphasizing active engagement, hands-on exploration, and open-ended questions to foster discussion and meaning making. Activities were designed to reinforce key sub-dimensions of mathematical reasoning through repeated and varied practice, promoting deeper cognitive processing and conceptual understanding. Accordingly, the study aimed to provide empirical evidence regarding the extent to which a structured, parent-mediated home intervention can contribute to the development of mathematical reasoning skills in early childhood.

## 2 Method

### 2.1 Research model

The study employed a true experimental research design, specifically a randomized pretest–posttest control group design, to examine the effectiveness of the Home-Based Mathematical Reasoning (HBMR) program on preschool children’s mathematical reasoning skills. True experimental designs are considered the strongest approach for establishing causal relationships because participants are randomly assigned to experimental and control groups, thereby minimizing selection bias and enhancing internal validity (Capili and Anastasi, 2024; Maciejewski, 2018). In the present study, children were randomly assigned to either the experimental group or the control group following pre-test assessments. The dependent variable was children’s mathematical reasoning skills, whereas the independent variable was participation in the HBMR program. The randomized pretest–posttest control group design enabled the researchers to evaluate the effectiveness of the intervention by comparing changes in mathematical reasoning skills across groups over time. The symbolic representation of the research model is presented in Table 1.

**Table 1:** Symbolic representation of the research model.

Group	Pre-test	Application	Post-test
Experimental Group	O₁	X (HBMR)	O₂
Control Group	O₃	—	O₄

O₁, O₃: Pre-test measurements (Mathematical reasoning skills), X: HBMR program application, O₂, O₄: Post-test measurements, —: No intervention was applied.

### 2.2 Study group

The study was conducted in the Eastern Anatolia region of Türkiye. This region was selected because, to the best of the authors’ knowledge, no previous home-based mathematics intervention studies targeting preschool children had been conducted in this geographical area. To ensure representation of different socioeconomic settings, ten independent public preschools serving children from different socioeconomic backgrounds were included in the study.

Participant selection was carried out in two consecutive stages. In the first stage, children who met the predefined eligibility criteria were identified within each preschool. The eligibility criteria were established based on the target population of the study and the intended population of the Mathematical Reasoning Skills Assessment Tool. Accordingly, only children who were between 60 and 72 months of age, demonstrated typical development, and had written informed consent provided by their mothers were considered eligible for participation. Because the number of eligible children differed across preschools, two children were randomly selected from the eligible pool of each preschool using a computer-generated random number procedure. This process resulted in a total sample of 20 children (two children from each of the 10 participating preschools). All selected children completed the pre-test assessment before group allocation.

In the second stage, following completion of the pre-test assessment, randomization was performed independently within each preschool using a computer-generated random allocation procedure. Of the two children selected from each preschool, one was randomly assigned to the experimental group and the other to the control group. Consequently, each of the ten participating preschools contributed one child to the experimental group and one child to the control group, resulting in two groups of ten children. This allocation procedure ensured that both groups were equally represented across the participating preschools and their respective socioeconomic settings. At both stages of the participant selection and allocation process, all randomization procedures were computer-generated, and the researchers had no influence on participant selection or group assignment.

Children assigned to the experimental group participated in the five-week home-based mathematics intervention together with their mothers. In contrast, children assigned to the control group continued their regular preschool education without receiving the intervention. No participant withdrew from the study during the intervention period, and all participants completed both pre-test and post-test assessments.

Pre-test analyses indicated no statistically significant differences between the experimental and control groups in terms of demographic characteristics or mathematical reasoning scores (*p* > 0.05), suggesting baseline equivalence between groups. In the experimental group, 50% of the children were girls and 50% were boys, and 70% had previously received preschool education. Regarding parental education, 50% of mothers held undergraduate degrees and 30% held graduate degrees, while 40% of fathers held undergraduate degrees and 40% held graduate degrees. In the control group, 40% of the children were girls and 60% were boys, and 70% had previously received preschool education. Among mothers, 40% held undergraduate degrees and 30% held graduate degrees, whereas 50% of fathers held undergraduate degrees and 20% held graduate degrees.

### 2.3 Data collection tools

The study utilized the “General Information Form” and the “Early Mathematical Reasoning Skills Assessment Tool” as data collection instruments.

*General Information Form:* This form was prepared to collect sociodemographic information about the children and mothers included in the study. In addition to information such as the child’s gender and preschool education history, the form also includes demographic data on the educational status of the mother and father.

*Early Mathematical Reasoning Skills Assessment Tool:* Developed by Ergül (2014), this assessment tool was specifically designed for typically developing children aged 5–6 years. It consists of 40 items organized into two sub-dimensions. The tool is administered through individual interviews with children. This assessment tool consists of 40 questions. Twenty-eight of these questions are accompanied by pictures; nine are accompanied by various materials, and the remaining three are asked verbally without any materials. Twenty-one questions of the measurement tool are in the measurement domain, while 19 questions are in the data analysis-probability domain. There are 21 questions in inductive reasoning and 19 questions in deductive reasoning. Item analysis conducted to determine how well the tool’s characteristics distinguish between children revealed a significant difference between the top 27% and bottom 27% groups. Accordingly, it was determined that the assessment tool is sufficient to distinguish between children with high and low mathematical reasoning skills (Ergül and Artan, 2015).

Regarding inter-rater reliability, the Krippendorff alpha coefficients for the 40 questions ranged from 0.73 to 1.00. The average alpha coefficient was 0.89. Test–retest reliability was found to be above 0.95 for all areas (Ergül and Artan, 2015). Cronbach’s alpha was used to evaluate the scale’s reliability. The alpha test provides information about the internal consistency of the scale. The reliability coefficients of the scale were found to be 0.76 for mathematical reasoning, 0.73 for induction, and 0.79 for deduction.

### 2.4 HBMR program development process

The program is based on the developmental characteristics of 60-72-month-old children and the needs of parents and society. Centered on Piaget's (1970) principle of concrete experience the program aims to support cognitive development. It is structured around constructivist principles (Çetinkaya, 2023) to promote active engagement and deeper cognitive processing consistent with higher-order thinking skills described in Bloom’s Taxonomy (Güven and Yaşartürk, 2025). At the same time, the program was designed using the Taba (1962) and Tyler (1969) program development model, and the systematic stages of program development were followed. Prior to the program, a needs assessment form was administered to five teachers to gather their opinions on the activities to be carried out. In the needs assessment form, teachers suggested activities related to mathematical reasoning, such as part-whole relationships, matching, sequencing, classification, time concepts, estimation, pattern formation, and number and operation skills. In this context, based on both the needs assessment form and the theoretical background, the main objective of this program is to develop children’s mathematical reasoning skills by implementing the HBMR program for children aged 60–72 months.

Since the main objective of the program is to make abstract mathematical concepts concrete for children, the researchers developed 20 different mathematical activity materials to be used in these activities.

#### 2.4.1 HBMR program implementation process

This pilot study, conducted to determine the effect of the HBMR program on the mathematical reasoning skills of preschool-aged children, involved a five-week intervention designed to yield preliminary findings. Before beginning the intervention, the researchers held introductory and orientation sessions with the teachers at the participating schools and the children who would take part in the study. However, teachers were not involved in the intervention or the assessment process. Additionally, an informational meeting was held for the mothers of children in the experimental group to explain the program’s purpose, content, and implementation process. After obtaining informed consent forms, home visits were scheduled with families in the experimental group; for parents of children in the control group, only written permission was obtained for participation in the pre-test and post-test assessments.

The home-based mathematics education program developed for this study was implemented in Turkish over a five-week period. All educational materials, parent guidelines, and activity contents used during the intervention were prepared and implemented in Turkish. The Turkish version of the Early Mathematical Reasoning Skills Assessment Tool, which was used for data collection, had previously undergone validity and reliability studies.

Two home visits were conducted each week for the 10 families in the experimental group, and a total of 10 sessions were held with each family. The visits were scheduled based on the families’ availability; however, to maintain the integrity of the intervention process, care was taken to conduct them on the same day and at the same times for each family whenever possible. Each session lasted approximately 40 min and consisted of two math activities. Thus, each child participated in a total of 400 min of the intervention process and completed 20 structured math activities. A total of 100 home visits were conducted during the program. The duration and session structure of the HBMR program are presented in Table 2.

**Table 2:** Implementation duration and session structure of the HBMR program.

Variable	Value
Total duration	5 weeks
Number of families	10
Weekly session	2
Total sessions (per family)	10
Session duration	40 min
Total intervention time (per child)	400 min
Total home visits (overall)	100
Total activities	20

To ensure intervention fidelity, all families followed the same sequence of activities within the same week, and the content was implemented according to a standardized flow. Prior to each session, the home environment was prepared to be suitable for conducting the activities, the necessary materials were gathered, and the implementation conditions were standardized as much as possible. To minimize interruptions during sessions, siblings did not participate in the intervention activities and, whenever possible, remained in another area of the home under the supervision of another family member.

During the implementation process, a second researcher assisted the principal investigator and observed whether the program was carried out as planned according to the prepared implementation checklist, whether the activity content was adhered to, and whether the implementation procedures were carried out in a standardized manner. While the active participation of the implementing researcher in the process is inevitable due to the nature of intervention studies, this was considered one of the study’s limitations. Similarly, due to the structure of the intervention, it was not possible for the researcher or the families in the experimental group to be blinded to group assignments. The weekly implementation schedule for the HBMR program is presented below. The program flow is presented below.

*First week:* Activities focused on the *matching* theme. During the first week, four activities were carried out, two in each session. These activities included matching shadow silhouettes to support children’s visual perception, matching animal pictures with their food to develop logical thinking, matching colors to reinforce basic knowledge, and matching numbers with objects to build mathematical concepts.

*Second week:* Activities were structured around *classification*. The second week featured four activities, with two activities per session. Within the scope of these activities, vehicles were classified according to where they move (in the air, on land, and in water), foods were classified into healthy and unhealthy groups, the relationship between the parts and wholes of objects was examined, and finally, objects were classified according to their shapes (square, rectangle, circle, and triangle).

*Third week:* The activities in the third week were structured around the theme *of comparison*. In the third week, four activities were conducted, two per session. In these activities, objects were compared for quantity (few-many), size (big-small), length (long-short), and weight (heavy-light).

*Fourth week:* The activities in the fourth week were structured around the theme of *sequencing*. In the fourth week, four activities were carried out, two in each session. These activities were carried out in the form of rhythmically sequencing numbers (numerical sequencing), sequencing events in the order they occurred (sequencing), sequencing objects from largest to smallest (size sequencing), and sequencing according to a pattern (logical rule sequencing).

*Fifth week:* The activities in the fifth week were structured with a focus on *numbers and performing operations*. A total of four different activities were carried out in the fifth week, with two activities in each session. Within the scope of these activities, children used concrete support by performing basic operations through finger counting, reinforced the relationship between numbers and quantities by marking the number corresponding to the number of objects, and learned the relationship between parts and wholes and number sequences by placing pizza slices according to the numbers written on them. The fourth activity was designed to lay the foundations for children’s mental addition and subtraction skills by completing objects to 10 (e.g., how many more apples are needed to make 10 from three apples). The content of the program developed in this context is presented in Table 3.

**Table 3:** Content of the HBMR program.

Week	Theme	Activity content	Mathematical reasoning dimension
First week	Matching	Shadow-silhouette matching, animal-food matching, color matching, number-object matching	Visual perception, establishing logical relationships, symbol–object matching
Second week	Classification	Sorting tools by movement area, classifying healthy–unhealthy foods, part–whole relationship, and classifying by shape.	Categorization, making conceptual distinctions
Third week	Comparison	Comparisons of less–more, big–small, long–short, heavy–light	Quantitative reasoning, measurement awareness
Fourth week	Sequencing	Rhythmic number sequencing, event sequencing, sorting by size, and pattern formation based on rules	Logical sequencing, pattern formation
Fifth week	Numbers and operations	Finger counting, number-quantity matching, pizza slice part-whole, completing to 10 activity	Numerical reasoning, mental preparation before operations

**Figure 1:**
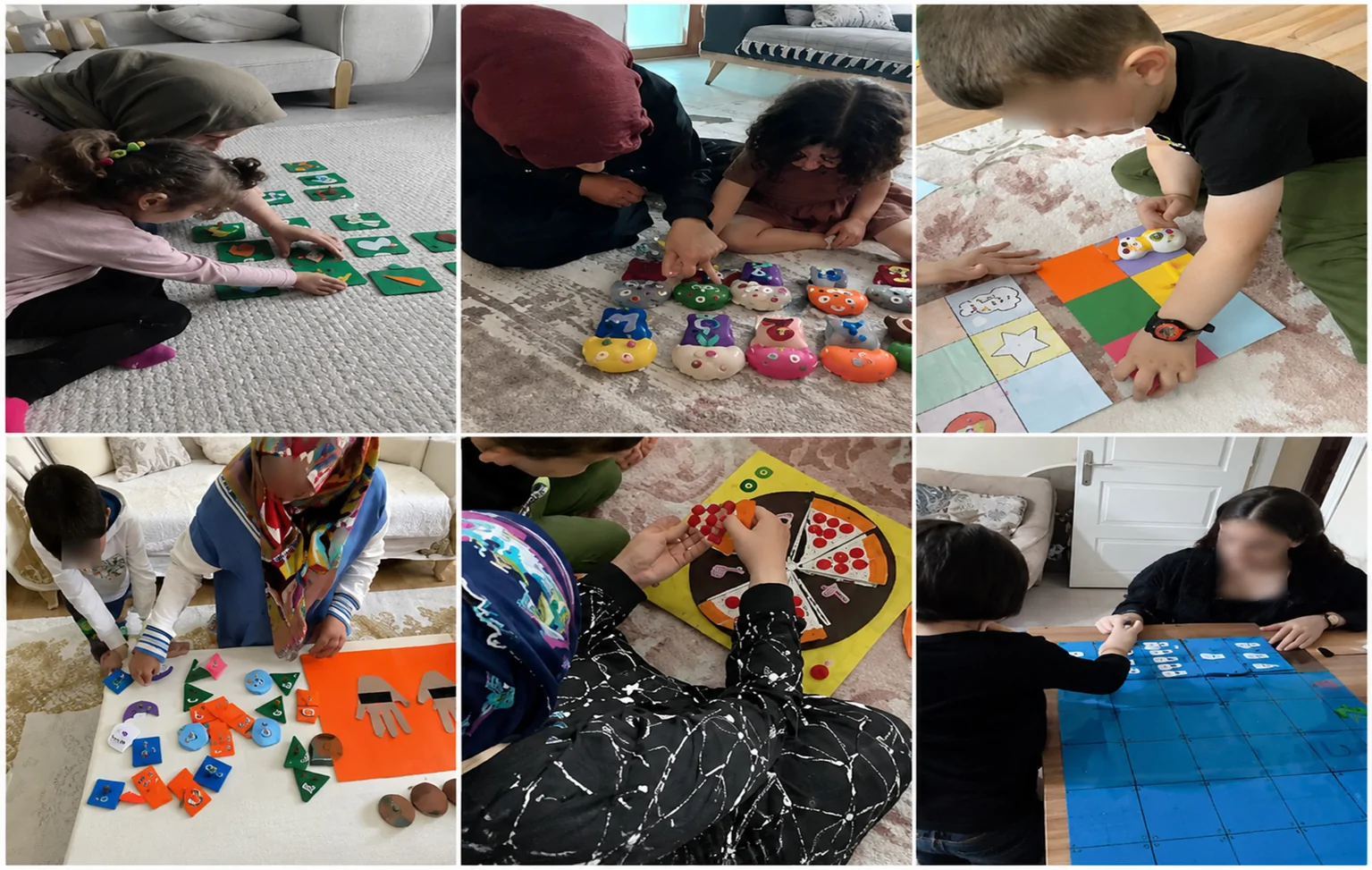
Visuals related to the HBMR program.

Visuals related to the HBMR program, implemented over ten sessions during 5 weeks, are presented in Figure 1.

### 2.5 Data collection

All necessary official and ethical approvals were obtained prior to the start of the study. First, the scientific and ethical appropriateness of this study was approved by the Erzincan Binali Yıldırım University Human Research Ethics Committee (Decision Date: May 28, 2025; Number: 05/02). In addition, protecting the rights of participants and ensuring their voluntary participation were of great importance. In this context, the Declaration of Helsinki was used as a basis. This declaration defines the protection and safeguarding of volunteers’ health, well-being, and rights as the fundamental duty of researchers.

#### 2.5.1 Data analysis

For data analysis, the responses of the experimental and control groups to the Early Mathematical Reasoning Skills Assessment Tool were entered into SPSS. Since the sample size in both groups was less than 30, the normality assumption was tested using the Shapiro–Wilk test, and skewness and kurtosis were examined. Skewness and kurtosis values between −3 and +3 are considered sufficient for normality (Büyüköztürk et al., 2015). Furthermore, the *p* < 0.05 criterion was used in significance tests. Since the data did not show a normal distribution, the Mann–Whitney U test was used to compare the groups for the pre-test and post-test. The Wilcoxon signed-rank test was used in the relevant groups to evaluate the difference between the pre-test and post-test scores within the experimental and control groups. Furthermore, the effect size was calculated using the formula r = Z/√N for non-parametric tests and interpreted according to Cohen's (1988) criteria.

## 3 Findings

The study aimed to evaluate the effect of the HBMR program on the mathematical reasoning skills of children aged 60–72 months. In this context, the children’s pre-test and post-test mean scores were examined. The findings obtained from the data are presented below.

Table 4 summarizes the descriptive statistics (mean, standard deviation, median, and minimum–maximum values) for the pre-test and post-test scores of the experimental and control groups. Although both groups displayed comparable baseline scores, the experimental group showed higher post-test scores across all outcome measures, whereas the control group showed only slight changes. The statistical significance of these differences is examined in the subsequent Mann–Whitney U and Wilcoxon signed-rank test results (Tables 5–9).

**Table 4:** Descriptive statistics of the pre-test and post-test scores for early mathematical reasoning skills in the experimental and control groups.

Sub-dimension	Group	*n*	Pre-test Mean ± SD	Median (Min–Max)	Post-test Mean ± SD	Median (Min–Max)
Measurement	Experimental	10	7.80 ± 6.18	5.5 (1–19)	9.90 ± 5.43	8.0 (3–19)
	Control	10	6.80 ± 4.92	6.5 (0–19)	7.00 ± 5.25	5.5 (3–21)
Data analysis	Experimental	10	13.90 ± 0.88	14.0 (12–15)	15.90 ± 1.10	16.0 (14–18)
	Control	10	13.80 ± 1.87	14.5 (10–16)	14.20 ± 1.62	14.5 (10–16)
Early mathematical reasoning skills	Experimental	10	21.70 ± 6.53	19.0 (15–34)	25.80 ± 5.35	24.0 (20–35)
	Control	10	20.60 ± 4.17	20.0 (15–31)	21.20 ± 5.14	20.0 (17–35)

**Table 5:** Pre-test scores for early mathematical reasoning skills and Mann–Whitney U test results for children in the experimental and control groups.

Sub-dimensions	Group	*n*	Mean rank	Sum of ranks	U	Z	*p*
Measurement	Experimental	10	10.10	101.00	46.000	−0.304	0.761
	Control	10	10.90	109.00			
Data analysis	Experimental	10	9.80	98.00	43.000	−0.531	0.595
	Control	10	11.20	112.00			
Early mathematical reasoning skills	Experimental	10	10.00	100.00	45.000	−0.379	0.705
	Control	10	11.00	110.00			

**Table 6:** Mann–Whitney U test results for the post-test scores of early mathematical reasoning skills in the experimental and control groups.

Sub-dimensions	Group	*n*	Mean rank	Sum of ranks	U	Z	*p*
Measurement	Experimental	10	12.55	125.50	29.500	−2.252	0.040
	Control	10	8.45	84.50			
Data analysis	Experimental	10	13.95	139.50	15.500	−2.617	0.009
	Control	10	7.05	70.50			
Early mathematical reasoning skills	Experimental	10	13.70	137.00	18.000	−2.421	0.015
	Control	10	7.30	73.00			

**Table 7:** Results of the Wilcoxon signed-rank test for dependent samples regarding the difference between the post-test scores and pre-test scores of the early mathematical reasoning skills test for children in the experimental group.

Sub-dimensions	Post-test-pre-test	*n*	Mean rank	Sum of ranks	Z	*p*
Measurement	Negative ranks	0	0.00	0.00	−2.684	0.007
	Positive ranks	9	5.00	45.00		
	Ties	1				
Data analysis	Negative ranks	0	0.00	0.00	−2.831	0.005
	Positive ranks	10	5.50	55.00		
	Ties	0				
Early mathematical reasoning skills	Negative ranks	0	0.00	0.00	−2.812	0.005
	Positive ranks	10	5.50	55.00		
	Ties	0				

**Table 8:** Results of the Wilcoxon signed-rank test for dependent samples regarding the difference between the post-test scores and pre-test scores of the children in the control group on the early mathematical reasoning skills test.

Sub-dimensions	Post-test-pre-test	*n*	Mean rank	Sum of ranks	Z	*p*
Measurement	Negative ranks	1	4.00	4.00	−0.378	0.705
	Positive ranks	3	2.00	6.00		
	Ties	6				
Data analysis	Negative ranks	0	0.00	0.00	−1.414	0.157
	Positive ranks	2	1.50	3.00		
	Ties	8				
Early mathematical reasoning skills	Negative ranks	1	2.00	2.00	−1.134	0.257
	Positive ranks	3	2.67	8.00		
	Ties	6				

**Table 9:** Effect sizes related to the pretest–posttest comparison of the experimental and control groups.

Group	Sub-dimensions	n	Z	p	r (Z/√N)	Bootstrap 95% CI	Effect level
Experimental	Measurement	10	−2.684	0.007	0.85	0.75–0.91	Large
	Data analysis	10	−2.831	0.005	0.90	0.89–0.92	Large
	Early mathematical reasoning skills (Total)	10	−2.812	0.005	0.89	0.89–0.91	Large
Control	Measurement	10	−0.378	0.705	0.12	0.00–0.70	Small
	Data analysis	10	−1.414	0.157	0.45	0.32–0.71	Medium
	Early mathematical reasoning skills (Total)	10	−1.134	0.257	0.36	0.00–0.71	Medium

Table 5 shows that when the pre-test scores of the experimental and control groups for early mathematical reasoning skills were compared using the Mann–Whitney U test, there was no significant difference between the experimental and control groups in terms of measurement, and no significant difference was found in terms of data analysis and early mathematical reasoning skills (*p* > 0.05). According to these findings, children in the experimental and control groups received similar scores in the pre-test phase before the application, indicating that the groups were equivalent.

Table 6 shows that when the post-test scores of the experimental group and the control group on early mathematical reasoning skills were compared using the Mann–Whitney U test, the results indicated significant differences in measurement, data analysis, and early mathematical reasoning skill scores (*p* < 0.05). To determine the source and direction of the difference in these findings, the mean ranks were examined. Accordingly, the children in the experimental group had larger early mathematical reasoning scores than the children in the control group. Based on these findings, children in the experimental group showed greater improvement in mathematical reasoning skills compared to the control group.

When the experimental group’s mathematical reasoning scores were examined using the Wilcoxon signed-rank test (Table 7), the post-test scores in both sub-dimensions and the total scale were significantly larger than the pre-test scores (*p* < 0.05).

When the control group’s mathematical reasoning scores were examined using the Wilcoxon signed-rank test (Table 8), no significant differences were found between the pre-test and post-test scores in either the sub-dimensions or the total scale (*p* > 0.05).

Table 9 presents the Wilcoxon signed-rank test effect sizes (r = Z/√N) for the pre-test–post-test comparisons of the experimental and control groups. Effect sizes were interpreted according to Cohen's (1988) criteria. Large effect sizes were observed in the experimental group for measurement (r = 0.85, 95% CI = 0.75–0.91), data analysis (r = 0.90, 95% CI = 0.89–0.92), and total early mathematical reasoning skills (r = 0.89, 95% CI = 0.89–0.91). These findings indicate that the HBMR program was associated with substantial improvements in mathematical reasoning skills within the experimental group. However, given the pilot nature of the study and the limited sample size, these effect sizes should be interpreted with caution and confirmed in larger randomized controlled trials.

In the control group, effect sizes were small for measurement (r = 0.12, 95% CI = 0.00–0.70), moderate for data analysis (r = 0.45, 95% CI = 0.32–0.71), and moderate for total early mathematical reasoning skills (r = 0.36, 95% CI = 0.00–0.71). Consistent with the Wilcoxon signed-rank test results, these changes were not statistically significant. Overall, the pattern of effect sizes suggests that the observed improvements were greater in the experimental group than in the control group, although these findings should be interpreted as preliminary because of the small sample size.

## 4 Discussion

This study was conducted over 5 weeks with 10 children aged 60–72 months attending independent preschools at different socioeconomic levels. In this context, the program was planned as two separate sessions per week, with two activities conducted in each session. In the study, the experimental group underwent the interventions, while the children in the control group did not undergo any interventions and continued their routine educational activities at independent preschools.

These findings are consistent with studies showing that mathematics education programs applied to preschool children can improve their reasoning skills (Balladares et al., 2024; Niklas and Schneider, 2017; Hallumoğlu et al., 2023). In this context, research conducted by Casey et al. (2018) found that applying mathematical reasoning skills to preschool children at an early age developed their mathematical reasoning skills and that these effects continued into first grade, supporting children’s academic achievement. Similarly, a study by Çelik and Kandır (2013) examined the effect of the “Big Math for Little Kids” educational program on the mathematical development of children aged 61–72 months. The results showed that the program was effective and improved the mathematical reasoning skills of children in the experimental group. Similarly, a study by Deleş et al. (2025) examined the effect of digital games on the development of preschool children’s mathematical skills. The study concluded that the activities and applications improved children’s mathematical skills.

The HBMR program was implemented as a home-based application. In this context, mothers were also encouraged to support their children’s reasoning skills in a structured manner. A review of the literature reveals that parents do not fully understand how to teach mathematics to their children (Ergel and Aydoğan, 2021), generally associate mathematics with process- and number-focused activities (Casey et al., 2018), and mostly repeat school homework at home (Puccioni, 2018). However, parents’ attitudes toward mathematics and their past experiences also influence their participation in math activities with their children (Bierman et al., 2016; Bilgen and Akman, 2021; DeFlorio and Beliakoff, 2015; Meyer et al., 2011; Mues et al., 2022). Recent evidence further suggests that caregiver-supported mathematical interactions, particularly those embedded in everyday home activities, can strengthen children’s mathematical language and early numeracy development, highlighting the importance of structured parental involvement in early mathematics learning (Purpura et al., 2021). In addition, learning trajectory research emphasizes that developmentally sequenced and intentional mathematical experiences are more effective when children are actively engaged in meaningful learning opportunities that are aligned with their current level of mathematical thinking (Clements and Sarama, 2021). In this context, it is thought that the home-based structure of the program enables mothers to become more familiar with their children’s developmental characteristics, strengthen their positive relationships with them, and receive support while preparing activities for their children. At the same time, research shows that home-based practices support children’s healthy development (Bachman et al., 2020; Çelik et al., 2025; Elbir, 2023; Gür and Kölemen, 2024; Hornburg et al., 2021; Panaoura, 2021; Sonnenschein et al., 2020; Tay et al., 2021). Furthermore, contemporary evidence indicates that early mathematical competence develops through the interaction of domain-general cognitive processes and domain-specific numerical skills, both of which can be supported through rich and purposeful mathematical experiences provided in children’s everyday environments (Mera et al., 2026). In this context, the meaningful effect of the HBMR program on children may be associated with the structured involvement of mothers during the home-based activities. However, since maternal participation was not directly measured in the present study, this interpretation should be considered cautiously.

The findings provide preliminary evidence that the home-based HBMR program may improve preschool children’s mathematical reasoning skills when implemented with maternal participation. Although effect sizes were observed (Table 9; *r* = 0.85–0.90), the pilot nature of the study and the small sample size warrant cautious interpretation of these findings. Therefore, the effectiveness of the HBMR program should be confirmed in larger, adequately powered randomized controlled trials. The results also suggest that structured, play-based mathematical activities implemented at home with parental involvement could contribute to the development of children’s mathematical reasoning skills. Nevertheless, given the pilot nature of the study and its limited sample size, the findings should be interpreted with caution and should not be generalized without replication in larger and more diverse samples. Furthermore, because the same assessment instrument was administered at both the pre-test and post-test over a five-week interval, the possibility of a practice effect resulting from repeated exposure to the assessment cannot be completely excluded. Although the control group showed only minimal changes in performance, this potential source of bias should be taken into account when interpreting the findings.

### 4.1 Practical findings and strengths

This study is a preliminary evidence study that provides important information to practitioners, families, and researchers by demonstrating that home-based structured math activities improve children’s mathematical reasoning skills. This study may support the idea that the home is the child’s most important learning environment and that children’s academic development can be supported at home through a structured program. Furthermore, the study shows that the guidance and financial support provided to parents enabled them to participate in their children’s learning process. This demonstrates the study’s potential to support equal opportunities in education, particularly as an alternative option for disadvantaged children or those with limited access to preschool education. At the same time, the design of easy-to-use, low-cost materials for each activity can facilitate the adoption and implementation of the program. Finally, the increase in quality time spent between mother and child is also a significant gain for families.

### 4.2 Limitations

This study was designed as a small-scale intervention and conducted with a limited sample size comprising an experimental group (*n* = 10) and a control group (*n* = 10). Therefore, caution should be exercised when generalizing the findings to larger populations. Although random selection and stratified random assignment procedures were employed to reduce selection bias and strengthen internal validity, participation depended on parental consent and voluntary participation. The intervention was delivered through individual home visits conducted by a researcher and an observer-researcher. A total of 100 home visits were completed over a five-week intervention period. The researchers’ presence during home-based activities may have influenced family participation and children’s motivation; therefore, some of the observed effects may partially reflect participants’ responses to being involved in the study. In addition, outcome assessments were not conducted under blinded conditions because the researchers involved in implementing the intervention also administered the assessment procedures. Consequently, the possibility of assessor bias should be taken into account when interpreting the findings. Furthermore, no formal correction for multiple comparisons was applied across the three outcome measures. Therefore, the possibility of Type I error inflation should be considered when interpreting the findings. Finally, because participants in the experimental and control groups were recruited from the same preschools, the possibility of contamination between groups cannot be completely ruled out. Although the intervention was implemented individually at home with mothers and intervention materials were provided only to families in the experimental group, informal interactions among children outside the intervention sessions may have occurred. Therefore, this potential source of contamination should be considered when interpreting the study findings.

## 5 Conclusion

In conclusion, this pilot randomized controlled trial demonstrates that the HBMR program can support mathematical reasoning skills in preschool children. However, given the study’s pilot nature, small sample size, and limited statistical power, these findings should be considered preliminary and interpreted with caution. Randomized controlled trials with larger, more diverse samples are needed to determine the effectiveness of the HBMR program and validate the current findings.

## Data Availability

The raw data supporting the conclusions of this article will be made available by the authors, without undue reservation.
