# Supplementary material for: The role of home-based math activities in developing mathematical reasoning skills in preschool children: a pilot randomized controlled trial
Source: Front Psychol. 2026 Sep 1;17:1903843. doi: 10.3389/fpsyg.2026.1903843 (PMC13574960; doi:10.3389/fpsyg.2026.1903843)
Supplement: Supplementary file 1 [file Supplementary_File_1.docx]

**Appendix 1. General Information Form**

**General Information Form**

This form was developed by the researchers to collect demographic information about the participating children and their families. The information obtained will be used only to describe the characteristics of the study sample and to examine participant characteristics in relation to the research findings. All information will be kept confidential and used exclusively for scientific research purposes. Data will be analyzed and reported only in aggregate form.

**A. Child Information**

1. Child's sex

☐ Female

☐ Male

2. Child's age (months)

__________ months

3. Has your child attended a preschool program before participating in this study?

☐ Yes

☐ No

If Yes, please indicate the duration of attendance.

☐ Less than 1 year

☐ 1 year

☐ 2 years

☐ More than 2 years

4. Has your child been diagnosed with any developmental, neurological, sensory, or physical disability?

☐ No

☐ Yes (Please specify): ______________________________

**B. Parent Information**

Mother

5. Educational attainment

☐ Elementary school

☐ Middle school

☐ High school

☐ Associate's degree

☐ Bachelor's degree

☐ Graduate degree

Father

6. Educational attainment

☐ Elementary school

☐ Middle school

☐ High school

☐ Associate's degree

☐ Bachelor's degree

☐ Graduate degree

C. Family Information

7. Person completing this form

.......

**Early Mathematical Reasoning Skills Assessment Tool**

| **INDUCTIVE REASONING ITEMS** | **MEASUREMENT**  **INSTRUCTIONS** | **Answers** | |
| --- | --- | --- | --- |
|  |  | **Wrong** | **Correct** |
| **1. Measuring with non-standard**  **Measuring with non-standard**  **(Length and weight)** | **1.** Can you arrange these strings in order of length? |  |  |
|  | **2.** Which one is the longest snake? **R-1** |  |  |
|  | **3.** Which person, wearing which pair of shoes, would take more steps if they measured this path? **R-2** |  |  |
|  | **4.** Which box is the heaviest? |  |  |
|  | **5.** Which animal is the heaviest? **R-3** |  |  |
|  | **6.** Which of the following can be placed on the other side of the scale to balance the pot?  **R-4** |  |  |
| **2. Measuring with non-standard**  **and comparing the results**  **comparing**  **(Area and volume)** | **7.** Which piece of paper requires the most paint? |  |  |
|  | **8.** All of these boxes are the same size and contain the same type of chocolate.  Which box contains the smallest chocolate pieces? **R-5** |  |  |
|  | **9.** To cover this wall, which of these stones should I use so that I’ll need the  stones will I need? **R-6** |  |  |
|  | **10.** Which box holds the most ping-pong balls? |  |  |
|  | **11.** To fill it completely, which pitcher do we need to add more water to?  Is it necessary? **R-7** |  |  |
|  | **12.** I have a lot of books. I can only place books of one color from each of the four types shown here  . Which type of book can the cabinet hold the most of? **R-8** |  |  |
| **3. Time**  **Order** | **13.** These candles were the same height when they were purchased. Which candle has been  longer? **R-9** |  |  |
|  | **14.** All of these pots are the same, and the same dish is cooking in each one. Which  dish will be ready first? **R-10** |  |  |
|  | **15.** All these buckets are the same. Which bucket fills up faster? **R-11** |  |  |
| **DEDUCTIVE REASONING**  **ITEMS** | **INSTRUCTIONS** |  | |
| **4. The one in hand**  **Verifying the accuracy of the results (testing)** | 1**6.** They measured this black line using the red sticks and found that it was 5 sticks long. Which  red rod do you think they used to arrive at this result? **R-12** |  |  |
|  | **17.** They placed these cubes from the first picture on the scales. Then they took the red cube  and placed it on the scale shown in the second picture. Which of these children is the lightest? **R-13** |  |  |
|  | **18.** The runners’ times were recorded using stopwatches. When the runners finished running, they stopped the stopwatches. Based on this,  which one came in first? **R-14** |  |  |
| **5. Verbal**  **comparison**  **problems** | **19.** When you walk through a muddy area, whose footprints—yours or your father’s—  footprints are deeper? |  |  |
|  | **20.** It’s raining heavily outside. To keep more people from getting wet, should you  an umbrella or a bus stop? |  |  |
|  | 2**1.** Does it take you longer to get home by bike or by car? |  |  |

1

| **INDUCTIVE ARGUMENT POINTS** | **DATA ANALYSIS AND PROBABILITY**  **GUIDELINES** | **Answers** | |
| --- | --- | --- | --- |
|  |  | **Wrong** | **Correct** |
| **1. Knowing the properties of shapes** | **22.** What are the common characteristics of these stamps? |  |  |
|  | **23.** What are the different characteristics of these buttons? **R-15** |  |  |
|  | **24.** Which shape is different from the others? **R-16** |  |  |
| **2. Creating a graph**  **Creation** | **25.** People at a bakery dropped a card into a box corresponding to the fruit juice they drank. Based on these cards, how can we show how many people  ? **R-17** |  |  |
|  | **26.** How can we place the shape tiles we have into these empty spaces? **R-18** |  |  |
|  | **27.** Look at the shapes on this page. How can we show how many of each shape there are in this  graph? **R-19** |  |  |
| **DEDUCTIVE REASONING**  **ITEMS** | **INSTRUCTIONS** |  | |
| **3. Examining the picture and predicting the situation depicted in the picture** | **28.** It’s raining very heavily outside. How many people might have just  ? **A-20** |  |  |
|  | **29.** How many people might have eaten at this table? **R-21** |  |  |
|  | **30.** Everyone living in this house has only one pair of shoes and one pair of slippers.  Since people going outside must wear shoes and those inside must wear slippers, how many people might be outside the house right now? **R-22** |  |  |
|  | **31.** How many people might be in this house right now? **R-23** |  |  |
| **4. Reading graphs and**  **state** | **32.** The children in a class looked at the weather and created this  graph using these cards. How many of each card did they use? **R-24** |  |  |
|  | **33.** Which house has the most people living in it? **R-25** |  |  |
|  | 3**4.** Using the shapes and small  pictures on the edge? **R-26** |  |  |
| **5. Probability**  **Statement** | **35.** I have 3 yellow balls and 1 green ball. Now I’m putting the balls into this bag  Let’s say I want to take a ball out of the bag. Which color ball is more likely to come out? |  |  |
|  | **36.** When I shoot an arrow while this circle is spinning quickly, which color is the arrow more likely to land on?  **R-27** |  |  |
|  | **37.** From which bag is the probability of selecting a red marble higher? **R-28** |  |  |
|  | **38.** What is the probability of rolling a number less than four when I roll the die? |  |  |
|  | **39.** What is the probability of rolling a 2 when I roll a die? |  |  |
|  | **40.** What is the probability of rolling a five when I roll the die? |  |  |

**ASSESSMENT AREA: INDUCTIVE REASONING SUBDIMENSION**

**FIGURE 1**

**FIGURE 2**

**FIGURE 3**

**FIGURE 4**

**FIGURE 5**

**FIGURE 6**

**FIGURE 7**

**FIGURE 8**

**FIGURE 9**

**FIGURE 10**

**FIGURE 11**

**MEASUREMENT AREA: DEDUCTIVE REASONING SUBDIMENSION**

**FIGURE 12**

**FIGURE 13**

**FIGURE 14**

**DATA ANALYSIS—PROBABILITY AREAS: INDUCTIVE REASONING SUBDIMENSION**

**FIGURE 15**

**FIGURE 16**

**FIGURE 17**

**FIGURE 18**

**FIGURE 19**

**DATA ANALYSIS—PROBABILITY SPACES, DEDUCTIVE REASONING SUBDIMENSION**

**FIGURE 20**

**FIGURE 21**

**FIGURE 22**

**FIGURE 23**

**FIGURE 24**

**FIGURE 25**

**FIGURE 26**

**FIGURE 27**

**FIGURE 28**

**Scoring Key for the Early Mathematical Reasoning Skills Assessment Tool**

| **Question**  **No** | **SCORE** | | | | | | **COMMENTS** |
| --- | --- | --- | --- | --- | --- | --- | --- |
|  | **0** | **1** | **2** | **3** | **4** | **5** |  |
| **1.** |  |  |  |  |  |  |  |
| **2.** |  |  |  |  |  |  |  |
| **3.** |  |  |  |  |  |  |  |
| **4.** |  |  |  |  |  |  |  |
| **5.** |  |  |  |  |  |  |  |
| **6.** |  |  |  |  |  |  |  |
| **7.** |  |  |  |  |  |  |  |
| **8.** |  |  |  |  |  |  |  |
| **9.** |  |  |  |  |  |  |  |
| **10.** |  |  |  |  |  |  |  |
| **11.** |  |  |  |  |  |  |  |
| **12.** |  |  |  |  |  |  |  |
| **13.** |  |  |  |  |  |  |  |
| **14.** |  |  |  |  |  |  |  |
| **15.** |  |  |  |  |  |  |  |
| **16.** |  |  |  |  |  |  |  |
| **17.** |  |  |  |  |  |  |  |
| **18.** |  |  |  |  |  |  |  |
| **19.** |  |  |  |  |  |  |  |
| **20.** |  |  |  |  |  |  |  |
| **21.** |  |  |  |  |  |  |  |
| **22.** |  |  |  |  |  |  |  |
| **23.** |  |  |  |  |  |  |  |
| **24.** |  |  |  |  |  |  |  |
| **25.** |  |  |  |  |  |  |  |
| **26.** |  |  |  |  |  |  |  |
| **27.** |  |  |  |  |  |  |  |
| **28.** |  |  |  |  |  |  |  |
| **29.** |  |  |  |  |  |  |  |
| **30.** |  |  |  |  |  |  |  |
| **31.** |  |  |  |  |  |  |  |
| **32.** |  |  |  |  |  |  |  |
| **33.** |  |  |  |  |  |  |  |
| **34.** |  |  |  |  |  |  |  |
| **35.** |  |  |  |  |  |  |  |
| **36.** |  |  |  |  |  |  |  |
| **37.** |  |  |  |  |  |  |  |
| **38.** |  |  |  |  |  |  |  |
| **39.** |  |  |  |  |  |  |  |
| **40.** |  |  |  |  |  |  |  |
